# Supplementation of Creatine Monohydrate Improves Sprint Performance but Has no Effect on Glycolytic Contribution: A Nonrandomized, Placebo-Controlled Crossover Trial in Trained Cyclists

**DOI:** 10.1016/j.cdnut.2025.104561

**Published:** 2025-01-28

**Authors:** Benedikt Meixner, Johanna Stegmaier, Peter Renner, Karsten Koehler, Woo-Hwi Yang, Billy Sperlich

**Affiliations:** 1 Department of Sport Science, Julius-Maximilians-Universität Würzburg, Integrative and Experimental Exercise Science & Training, Würzburg, Germany; 2 Department of Sport Science and Sport, Friedrich-Alexander-Universität Erlangen-Nürnberg, Erlangen, Germany; 3 iq-move Praxis Fraunberger, Erlangen, Germany; 4 Department of Health and Sport Science, Technical University of Munich, TUM School of Medicine and Health, München, Germany; 5 Graduate School of Sports Medicine, CHA University, Gyeonggi-do, Republic of Korea; 6 Department of Medicine, General Graduate School, CHA University, Gyeonggi-do, Republic of Korea

**Keywords:** alactic, high-intensity cycling, ergogenic aid, body composition, anaerobic, lactate, fat-free mass

## Abstract

**Background:**

Sprint performance may be crucial for athletes in sprint and endurance sports. In this regard, the maximal glycolytic rate (νLa_max_) is a variable within Mader’s model of metabolism that is commonly tested in a 15-s all-out sprint test. The product of lactate accumulation (ΔLa) and lactate distribution space approximated by fat-free mass (FFM) is strongly linked to sprint performance. Creatine monohydrate is a widely used ergogenic aid known for increasing anaerobic performance and FFM and the phosphagenic system contributes most to a 15-s all-out sprint.

**Objectives:**

The aim of the study was to analyze the influence of creatine supplementation of 15-s work, FFM, and ΔLa.

**Methods:**

Twenty male and 5 female cyclists underwent a placebo-controlled, crossover design with 4 laboratory visits. After a familiarization trial, baseline measurements were performed. Placebo (4 × 5 g/d maltodextrin) and creatine monohydrate (4 × 5 g/d) were administered for 5 d before the respective trials. All participants underwent this order of trials and performed a 15-s all-out sprint test on a Cyclus2-ergometer. Capillary blood was sampled before and every minute (for 8 min) after the sprint to determine ΔLa. Body composition was determined employing bioelectric impedance analysis.

**Results:**

Creatine supplementation significantly increased 15-s work output and FFM compared with baseline and placebo conditions. However, no significant differences were found in capillary blood lactate accumulation (ΔLa) or glycolytic contribution.

**Conclusions:**

The results indicate potential differences in limitations of sprint performance of participants. Responders to creatine supplementation are potentially limited by mechanical or metabolic factors. The findings of this study highlight the importance of considering supplementation of creatine monohydrate when undergoing testing for the maximal glycolytic rate.

## Introduction

Performance in sprint cycling is influenced by various factors, including the maximal glycolytic rate of muscle cells responsible for producing adenosine triphosphate (ATP) required for muscle contraction [1,2]. Directly assessing the maximal glycolytic rate in active muscles during sprint cycling is currently unfeasible. Instead, resting and peak post-exercise capillary blood lactate levels, measured after a 15-s sprint, serve as indirect proxy of this rate [[3], [4], [5], [6]]. One parameter derived from these measurements, known as νLa_max_, is considered a surrogate marker for the maximal glycolytic rate.

νLa_max_ has gained recognition as an indicator of metabolic performance in both endurance and sprint cycling, offering valuable insights into a cyclist’s glycolytic power and overall metabolic profile [1]. By estimating the maximal glycolytic rate, νLa_max_ assist to understand how quickly an athlete produces glycolytic energy, which is crucial for maintaining high power outputs during intense efforts and extended events [1,3]. Also, νLa_max_ aids in tailoring training and nutritional strategies to optimize performance by balancing anaerobic and aerobic demands [1], providing coaches and sports scientists with valuable information to enhance endurance performance. The 15-s all-out sprint that is employed in the determination of νLa_max_ [[7], [8], [9], [10], [11]] predominantly relies on the phosphagen and glycolytic pathways for energy production [7,[12], [13], [14]]. The phosphagen pathway, which utilizes ATP and phosphocreatine (PCr), delivers the highest energy flux rate. However, its limited capacity, because of small substrate reserves, restricts its sustainability [7]. The creatine kinase reaction (Formula 1) acts as a temporal buffer to when ATP demand is high and also acts as a buffer to protons as a byproduct of ATP hydrolysis [[15], [16], [17]].

$$ATP+Cr↔PCr+ADP+H^{+}$$

In contrast, the glycogenolytic and glycolytic pathway generates energy by converting glycogen and glucose into pyruvate and lactate. In this context, phosphofructokinase (PFK) activity serves as the rate-limiting factor in this pathway [18,19] and becomes further constrained during the sprint because of increased hydrogen ion accumulation from elevated ATP hydrolysis and decreased pH levels [20].

Recent studies [7,21] determined the energetic contribution of the phosphagen, glycolytic, and aerobic pathways to a 15-s all-out sprint. For this type of indirect assessment, phosphagen contribution is determined through gas exchange measurements, including *Off* $\dot{V}$ O_2_ kinetics, whereas glycolytic contribution is inferred from changes in capillary blood lactate concentration [7]. Especially glycolytic contribution is closely linked to power output during the 15-s all-out sprint test [22]. Phosphagenic sources contribute more than half of the energy in a 15-s all-out sprint [7].

The method for indirectly calculating glycolytic energy contribution is based on Margaria et al.’s original work with an accumulation of 1 mmol/L of capillary blood lactate corresponding to an energy equivalent of 3 mL oxygen per kg of body mass (BM) [13,23,24]. Building on this work, Mader and Heck [4] suggested that a more accurate measurement of lactate distribution space should consider fat-free mass (FFM) instead of total BM. In fact, this anthropometric adjustment enhances the calculation for glycolytic contribution, resulting in a more accurate energy equivalent for lactate accumulation [22].

Creatine monohydrate is a widely used nutritional ergogenic aid among athletes, primarily to enhance muscular power [25]. Predominantly stored in muscles, especially type II fibers [26], and typically sourced from animal foods, creatine supplementation has consistently been shown to increase intramuscular PCr stores [25,27]. This increase in PCr is considered the main mechanism for elevating anaerobic energy capacity [25].

Despite the strong scientific consensus on its efficacy as an ergogenic aid, so far no adverse health risks have been identified with creatine supplementation [25]. However, a common side effect of short-term creatine loading protocols is transient BM gain because of water retention [27,28]. This increase in FFM resulting from creatine supplementation also affects lactate distribution space [4,22].

Creatine supplementation may influence capillary blood lactate accumulation during a 15-s all-out cycle sprint by increasing phosphagen energy contribution. Furthermore, variations in capillary blood lactate concentrations could be linked to an expanded lactate distribution space, although these effects are likely minimal. Because creatine supplementation affects the distribution space of blood lactate, the aim of the present experiment was to determine the influence of creatine supplementation on *1*) 15-s work, *2*) capillary blood lactate accumulation, and *3*) glycolytic energy contribution, as calculated by the product of ΔLa and body composition, in a 15-s all-out sprint.

We hypothesized that creatine supplementation would *1*) increase 15-s work, *2*) reduce ΔLa because of the enlargement of lactate distribution space induced by creatine supplementation, and *3*) reduce glycolytic contribution compared with baseline testing and the placebo condition.

## Methods

### Participants

Twenty male and 5 female trained cyclists and triathletes were recruited for inclusion in this study. Sixteen participants self-identified as omnivores, 8 as vegetarians, and 1 as vegan. Originally, we aimed for 30 participants to detect a minimal effect size of 0.5 and to account for a dropout rate of 10%. Twenty-five participants were included in data analysis because 5 participants could not finish the required trials in the given timeframe for reasons unrelated to this study. As previously suggested, all participants were required to abstain from creatine supplementation in the 4 wk before study participation [29]. All participants were experienced road cyclists who use clipless pedals; additionally, they had extensive experience performing sprints in training and/or competition. Before the study, participants were informed of the protocol and provided written informed consent to participate. All procedures were approved by the Ethical Committee of Exercise Science & Training of the Faculty of Human Sciences of the University of Würzburg (EV2024/1-1004) and conducted in accordance with the Declaration of Helsinki [30]. Participants’ characteristics are given in Table 1. All data were collected between November 2023 and February 2024.

**TABLE 1:** Mean ± SD age, body stature, selected anthropometric data, and peak oxygen uptake of participants.

Variable	All (*n* = 27)
Age (y)	30.9 ± 8.4
Height (cm)	177.7 ± 6.4
Body mass (kg)	72.4 ± 9.1
Body fat (%)	13.1 ± 4.5
Fat-free mass (kg)	62.5 ± 8.1
Peak power (W)	1131 ± 237
Peak power (W/kg)	15.8 ± 4.0
Cycling (km/y)	9011 ± 4964

### Study design

**FIGURE 1:**
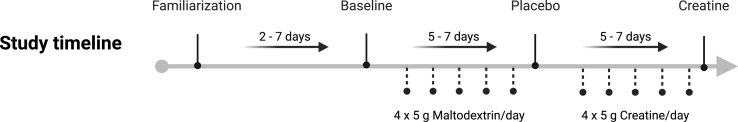
Timeline of study design (created with BioRender).

Participants completed 4 visits to the laboratory, each ≥48 h apart, within a period of 3 wk. Figure 1 illustrates the timeline and all testing procedures for each visit.

The study employed a placebo-controlled, sequential/nonrandomized crossover design as typically performed with this type of supplementation [31]. Because of the fixed order of conditions, only the participants were blinded to the study design. The fixed nonrandomized order was chosen because the relatively long washout period of creatine [32,33] could potentially influence the results because of changes in nutrition, training, or body weight. When a familiarization session is performed beforehand, the reliability of 15-s sprint and ΔLa is excellent, with an intraclass correlation coefficient >0.9 [34].

All visits to the laboratory involved the same experimental procedures described below. The data from the initial laboratory visit were excluded from the statistical analysis because it served as a familiarization trial. The first visit (T1) was designated as the baseline trial. In the 5 d preceding the second trial (T2), all participants were instructed to consume 4 daily doses of 5 g of maltodextrin (Alzchem GmbH) as a placebo [35].

In the 5 d preceding the third trial (T3), all participants were instructed to consume 4 daily doses of 5 g of creatine monohydrate (Creapure®, Alzchem GmbH) [36,37]. Participants were instructed to completely dissolve the contents of the powder sticks they received into a noncaffeinated liquid and to keep this liquid the same for the duration of the study. Figure 1 illustrates the study timeline.

### Experimental procedures

All participants were instructed to keep a nutrition diary and to follow their usual diet within the 24 h before each visit [38]. Additionally, on the day of the visit to the laboratory, all participants were instructed to remain adequately hydrated, consume a carbohydrate-rich meal (for example, a banana and a jam sandwich) no <3 h before each visit, and refrain from caffeine consumption.

For each visit, body composition, including FFM and BM, was measured using 8-electrode segmental multifrequency bioelectrical impedance analysis (1, 5, 50, 250, 500, 1000 kHz; InBody 720, InBody Co Ltd). All cycle sprints were conducted on a Cyclus2 ergometer (RBM elektronik) using the participants' personal road bikes. The Cyclus2 is an electromagnetically braked ergometer and measures power with an accuracy error of 2%, according to the manufacturer [34]. All cyclists used their own shoes and clipless pedals for all tests. During all 4 visits, cyclists warmed up for 10 min by cycling at 1.5 W/kg BM, followed by a 3-min rest [34].

The all-out cycle sprint was performed in a seated position using the large chainring (if applicable) of the participant’s bike and the 15-tooth cog of the ergometer. The recording of the test started once the cadence exceeded 30 RPM as per ergometer settings. Hand position was freely chosen by the participants but a grip on the drops was recommended. The position of the hands was kept constant throughout all visits. The ergometer software was set to isokinetic mode at 130 RPM [8,11,34,39].

**FIGURE 2:**
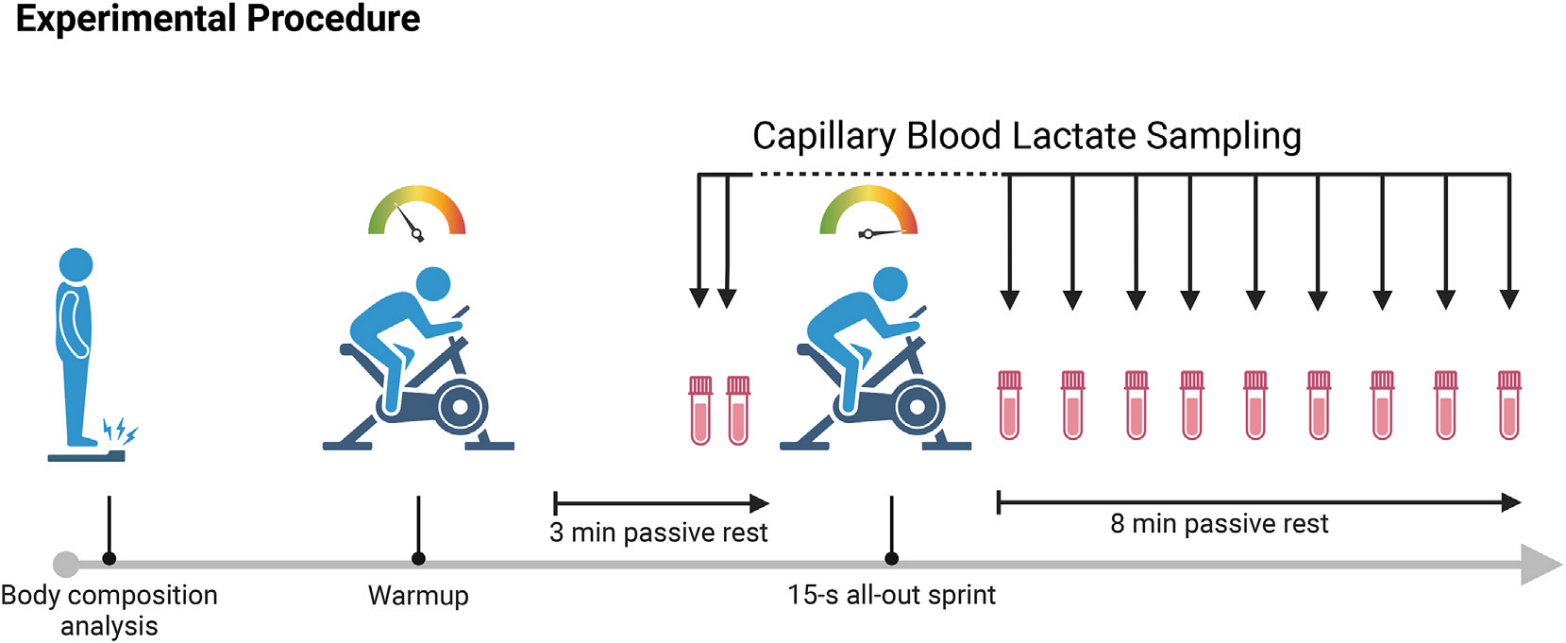
Timeline of experimental procedure during visit to the laboratory (created with BioRender).

Capillary blood samples were taken from the left earlobe twice during the resting period, after the warm-up, immediately after the sprint, and then every minute for 8 min after the 15-s cycle sprint. For the determination of lactate concentration, capillary blood samples (20 μL) were collected from the earlobe into an end-to-end capillary (20 μL, EKF Diagnostic). Immediately after collection, the filled capillary was mixed with a hemolyzing solution. Blood lactate concentrations were measured amperometric-enzymatically using the Biosen C-Line (EKF Diagnostics). The timeline of experimental procedures is shown in Figure 2.

### Glycolytic contribution

The change in blood lactate concentrations (ΔLa) was determined by subtracting the mean resting capillary blood lactate concentration from the peak post-exercise capillary blood lactate concentration. The calculation of glycolytic contribution was based on the method described by Margaria et al. [13,24]. Given that the lactate distribution space is ∼50% of FFM [4,40], we assumed 7 mL oxygen per mmol of capillary blood lactate accumulation per kg of distribution space instead of 3 mL oxygen per kg of BM [22].

### Responder analysis

Individual responder analysis is essential for understanding variability, optimizing training, and accurately interpreting the effects of creatine supplementation on performance and metabolic pathways. In our study, creatine monohydrate supplementation was expected to produce 1 of 2 outcomes when compared with baseline and placebo: either increased 15-s work for the same glycolytic contribution or reduced glycolytic contribution for equivalent 15-s work. To assess these changes, the smallest worthwhile change (SWC) was calculated by multiplying the between-subject SD of baseline measurements by 0.2, representing the smallest effect size of interest [11,[41], [42], [43]]. Nonresponders were identified when neither 15-s work nor ΔLa changed more than SWC under the creatine condition.

### Statistical analyses

Mean, SDs, and 95% confidence intervals (CIs) were computed using Microsoft Excel. Statistical analyses were conducted with GraphPad Prism (10). Data normality was assessed using the Shapiro–Wilk Test without requiring further transformation.

Differences between conditions were analyzed employing repeated-measures analysis of variance and post hoc multiple comparison Tukey test. No sphericity was assumed and Greenhouse-Geisser correction was applied. Equivalence between conditions was additionally tested employing the two 1-sided tests procedure [44,45] in jamovi (2.5.1.0, the jamovi project). Upper and lower bounds were set to standardized effect sizes of Cohen’s *d* of 0.5 and −0.5. Effect sizes were calculated as Cohen’s *d* [43].

## Results

All mean ± SD data for variables assessed during the 15-s cycle sprints are summarized in Table 2. Both 15-s work and FFM increased significantly with creatine supplementation but remained statistically equivalent under placebo treatment compared with baseline. ΔLa showed statistical equivalence under the placebo condition, but no differences or equivalence were observed for the creatine condition compared with baseline. The oxygen equivalent of lactate accumulation remained consistent across all measurements. The 15-s work relative to FFM increased slightly but did not reach statistical significance. Effect sizes, calculated as Cohen’s *d*, for various conditions are summarized in Table 3.

**TABLE 2:** Mean ± SD of variables during baseline, placebo, and creatine conditions.

Variable	At baseline	Placebo condition	With creatine
15-s work (J)	12,973 ± 183	12,910 ± 2662^1^	13,330 ± 2780^2^
15-s work/FFM (J/kg)	205.2 ± 30.5	204.3 ± 27.8^1^	207.8 ± 29.4
FFM (kg)	62.9 ± 7.9	62.9 ± 8.4^1^	63.9 ± 8.5^2^
BM (kg)	72.4 ± 9.3	72.2 ± 9.3	72.8 ± 9.4^1^
ΔLa (mmol/L)	8.66 ± 2.37	8.59 ± 2.25^1^	8.54 ± 2.32
Oxygen equivalent of W_Gly_ (mLO_2_)	3846 ± 1236	3823 ± 1206^1^	3862 ± 1281^1^

Abbreviations: ΔLa, difference between resting and peak post-exercise capillary blood lactate levels; BM, total body mass; FFM, fat-free mass; W_Gly_, glycolytic contribution to 15-s work.

1 Denotes statistical equivalence to baseline values.

2 denotes statistical difference to baseline values.

**TABLE 3:** Cohen’s *d* for relevant variables between baseline, placebo, and creatine conditions.

Variable	Cohen’s *d* Baseline – Placebo	Cohen’s *d* Baseline – Creatine	Cohen’s *d* Placebo – Creatine
15-s work (J)	0.119	−0.550	−0.944
15-s work/FFM (J/kg)	0.091	−0.229	−0.481
FFM (kg)	0.012	−0.643	−0.906
ΔLa (mmol/L)	0.094	0.165	0.055
Oxygen equivalent of W_Gly_ (mL oxygen)	0.058	−0.043	−0.103

Abbreviations: ΔLa, difference between resting and peak post-exercise capillary blood lactate levels; FFM, fat-free mass; W_Gly_, glycolytic contribution to 15-s work.

**FIGURE 3:**
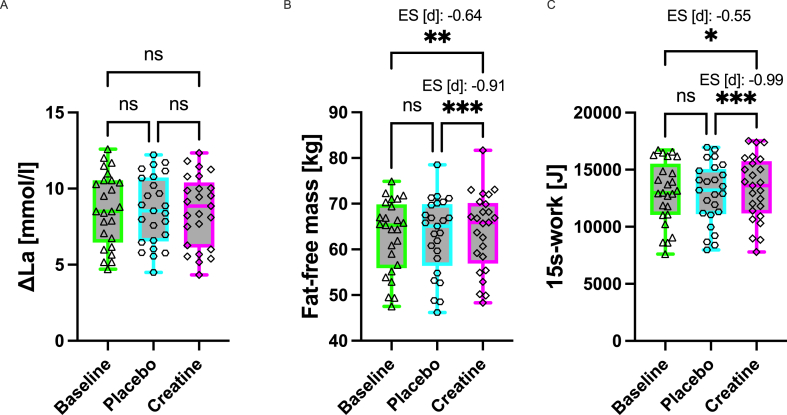
Individual and box-and-whisker-plot of baseline, placebo, and creatine conditions for (A) ΔLa, (B) fat-free mass, (C) 15-s work. ES, effect size (Cohen’s *d*); ΔLa, difference between resting and peak post-exercise capillary blood lactate levels. ∗/∗∗/∗∗∗level of significance at *P* < 0.05/0.01/0.001; ns, nonsignificant.

**FIGURE 4:**
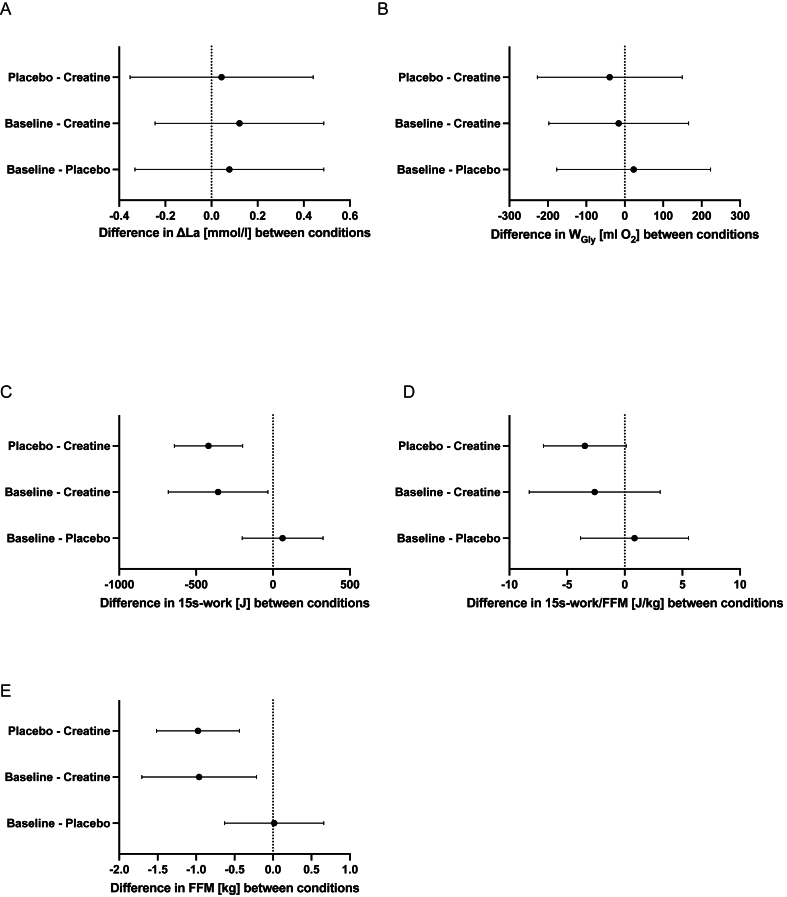
Comparison of differences between conditions and 95% CI for (A) ΔLa, (B) W_Gly_, (C) 15-s work, (D) 15-s work/FFM, (E) FFM. ΔLa, difference between resting and peak post-exercise capillary blood lactate levels; FFM, fat-free mass; W_Gly_, glycolytic contribution to 15-s work.

Individual results with differences between conditions are displayed in Figure 3. Mean differences between conditions along with the 95% CI are displayed in Figure 4.

**FIGURE 5:**
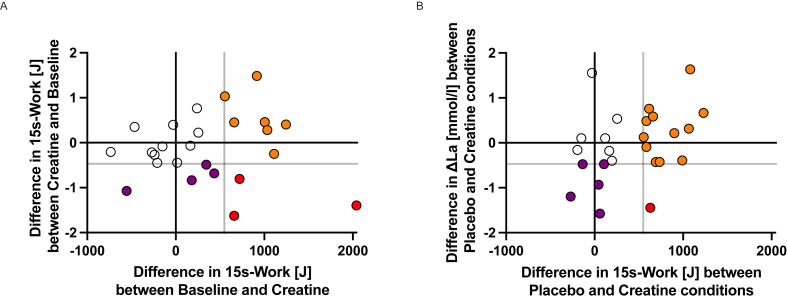
Individual differences between creatine supplementation and (A) baseline and (B) placebo condition. Responders are marked purple when ΔLa decreased more than SWC or orange when 15-s work increased more than SWC. When both conditions were met, responders are marked red. ΔLa, difference between resting and peak post-exercise capillary blood lactate levels; SWC, smallest worthwhile change.

SWC was calculated to ±0.47 mmol/L for ΔLa and ±548 J for 15-s work. Individual analysis of responders is displayed in Figure 5A and B.

## Discussion

This study aimed to assess the influence of creatine monohydrate supplementation on a 15-s all-out sprint test performance, FFM, and ΔLa. Compared with baseline testing and placebo condition, supplementation of creatine monohydrate *1*) increases absolute 15-s work output, but not significantly when adjusted for FFM, *2*) has an undetermined effect on ΔLa, and *3*) does not affect glycolytic contribution.

### 15-s work

Creatine supplementation has consistently been shown to enhance anaerobic performance [28,46]. Bundle and Weyand [2] argue that, unlike endurance exercise, sprint performance is not limited by metabolic factors but by the ability to apply musculoskeletal force. However, creatine supplementation increases intramuscular creatine stores [25,27,28], hereby enhancing the availability of anaerobic phosphagen energy reserves. This mechanism offers a metabolic explanation for the observed increase in 15-s work output associated with creatine supplementation, which provides a contrasting perspective to the previously mentioned viewpoint. Although increased PCr levels provide a focal point for the explanatory mechanism of increased anaerobic performance, other mechanisms might play an additional role: creatine supplementation can improve calcium kinetics in the sarcoplasmic reticulum [[47], [48], [49]] thereby reducing fatigue. PCr can also act as a buffer for protons during ATP hydrolysis [16]. Increased intramuscular buffering capacity via increased creatine content could allow for delayed inhibition of PFK [18]. Additionally, creatine supplementation leads to increased glycogen storage, potentially increasing the capacity of this pathway [50].

Studies have shown that neuromuscular function is positively influenced by creatine supplementation [51]. However, it is important to note that performance enhancement is not solely independent of metabolic energy demands.

Muscle fiber type distribution has been shown to play a role in responding to creatine supplementation [52]. In the absence of muscle biopsies, relative 15-s work could function as an indicator for a greater fraction of type II fibers. However, no such significant relation between increased 15-s work under the creatine condition and relative 15-s work was found in our data.

Creatine supplementation, particularly when administered through a loading protocol [27,28], increases FFM, which is primarily because of water retention in skeletal muscles [27]. The increase in FFM and body weight might contribute to the generation of greater forces during sprinting because of increased assistance of gravity on mechanical propulsion.

The rise in FFM and BM contributes to the generation of greater forces during sprinting. Although initially considered an adverse effect, the increased creatine stores and associated water retention elevate pressure within muscle compartments [53,54]. This rise in intrafascial pressure is potentially another mechanism that may enhance performance through increased stiffness and muscle tension.

### Effect on fat-free mass and lactate distribution space

When administered in a loading protocol with multiple doses of creatine monohydrate per day, an increase in FFM because of water retention can be expected [27,28]. The increase in FFM has 2 important implications for this study. First, as previously mentioned, a higher FFM facilitates higher power outputs [22]. Second, although there is a trend toward increased 15-s work relative to FFM with creatine supplementation, this trend did not reach statistical significance. As described by Mader and Heck [4], lactate distribution space is a fraction of FFM. Therefore, as FFM increases, the lactate distribution space also expands proportionally [4,22]. This expansion means that the same total amount of lactate produced is more diluted, theoretically leading to lower concentrations of capillary blood lactate.

### Effect on ΔLa and glycolytic contribution

Our results show no significant differences in ΔLa between the conditions. However, when testing for statistical equivalence, we found equivalence only between the baseline and placebo conditions, not between the baseline and creatine conditions. Therefore, we conclude that there might be a trend toward decreased lactate concentrations, but our sample size is insufficient to detect a significant difference between conditions.

The oxygen equivalent of lactate enables the determination of the glycolytic contribution during sprint tests [7] and accounts for FFM as lactate distribution space [22] as well as ΔLa. Traditionally, this calculation is based on BM as a contributing factor [13,14,24], but may be improved by considering FFM instead of total BM.

When considering glycolytic contribution, statistical equivalence was found between all analyzed conditions because of increased FFM and decreased ΔLa. On the basis of these findings, we conclude that the same amount of lactate is produced during the sprint test under all conditions. At the group level, creatine supplementation does not reduce glycolysis and lactate production but may slightly dilute lactate concentrations.

A previous study [55] determined the effect of nutritional interventions on ΔLa in a 15-s all-out tests. Unfortunately, only effects in ΔLa were described and actual performance effects remain unclear. Our study synthesizes the effects of a nutritional intervention in a comparable testing setup on 15-s work an ΔLa.

### Individual response

For this study, we identified responders to creatine supplementation during testing in 2 ways: a decrease in ΔLa larger than the SWC of 0.47 mmol/L, or an increase in 15-s work of 548 J. Among our 25 participants, 7 showed no changes larger than the SWC in either variable, 6 exhibited a decrease in ΔLa larger than the SWC, and 13 demonstrated an increase in 15-s work larger than the SWC (with 1 participant responding in both variables, see Figure 5A) when comparing placebo and creatine conditions. The results are similar when comparing baseline and creatine conditions (see Figure 5B).

A change in ΔLa is noteworthy because *1*) it is the primary parameter for calculating νLa_max_, and *2*) we identified differences between conditions in glycolytic contribution calculations as mainly related to differences in ΔLa.

The 3 responders who demonstrated a decrease in glycolytic contribution alongside an increase in 15-s work output (Figure 5A) were male and omnivorous. This observation aligns with existing literature, which remains inconclusive on whether vegetarians derive greater benefits from creatine supplementation compared with omnivores [56]. Furthermore, previous research has found that males tend to exhibit increases in anaerobic capacity after creatine supplementation, whereas females often do not [57]. This disparity may be attributed to the higher proportion of type II muscle fibers in males, a factor known to influence responsiveness to creatine supplementation [52].

A decrease in ΔLa indicates that phosphagen energy contribution replaces glycolysis during the sprint test, altering the sprint metabolism. Individuals with this response may be limited in their power output not by glycolytic energy contribution but rather by neuromuscular or mechanical factors such as the testing setup [2,22].

Increased 15-s work is the expected outcome of creatine supplementation. Although our methodology does not resolve the mechanism, increased creatine stores provide more phosphagen energy contribution. This is noteworthy in the context of our testing procedure, as we previously demonstrated a linear relationship between glycolytic contribution and 15-s work [22]. The gradient of this relation changes for individual responders under creatine supplementation.

### Strength and limitations

We consider the training status of our participants a strength of this study because we included only trained cyclists with competitive experience. Additionally, participants were experienced in sprint cycling in training and/or competition. The familiarization before baseline testing further improves the quality of our results because reliability improves when a familiarization is undertaken [8,34]. Additionally, the use of the participants own bike and shoe/pedal interface and the employment of an ergometer capable of isokinetic mode are important requirements for this testing procedure [58].

Measurements of oxidative and phosphagen energy contribution measured by gas exchange might allow for further insights into this matter but was unavailable at the time in the required measurement frequency [7].

We were unable to randomize placebo and creatine conditions caused by the relatively long washout period of creatine [33]. However, the high reliability of our testing procedure after a familiarization trial [34] combined with the blinding of participants allowed valid conclusions despite the sequential, nonrandomized study design. However, the potential risk of unblinding because of side effects, such as BM gain from water retention or differences in taste and solubility, remains a concern.

Our study could be improved by determination of intramuscular creatine levels before and after supplementation either by D_3_Creatine [57] or muscle biopsies. Muscle biopsies would further allow for the determination of muscle fiber distribution [52] and intramuscular lactate concentrations, providing further mechanistic explanations.

The study may lack sufficient power to detect a significant effect of 15-s work relative to FFM. Moreover, using a different dosage of creatine monohydrate or altering the intake protocol could influence the results. Specifically, a protocol without a loading phase but with consistent creatine supplementation over an extended period might lead to different outcomes, particularly in terms of changes in FFM and lactate distribution space.

### Conclusion

Creatine monohydrate supplementation enhances absolute 15-s work output, although this effect is not significant when adjusted for FFM. Although the impact on ΔLa remains inconclusive, creatine supplementation does not influence glycolytic contribution. Given these findings, when performing tests such as νLa_max_ in conjunction with 15-s sprints, consideration of creatine supplementation is important. The glycolytic contribution, as measured by the product of ΔLa and FFM, may serve as a more reliable indicator for assessing glycolytic capacity across participants.

## Author contributions

The authors’ responsibilities were as follows – BM, JS, PR, BS: conceptualization, methodology; BM, JS: investigation, formal analysis; BM, BS: writing—original draft; KK, PR, W-HY: writing—review and editing; BS: supervision; and all authors: read and approved the manuscript and the final version.

## Funding

Creatine (Creapure®) and placebo (maltodextrin) were provided by Alzchem Trostberg GmbH (Trostberg, Germany) free of charge. Alzchem Trostberg GmbH and its employees had no influence on the study protocol, statistical analysis, interpretation of results, and writing of this manuscript.

## Conflict of interest

The authors report no conflicts of interest.
